# Assessment of online patient education materials designed for people with age-related macular degeneration

**DOI:** 10.1186/s12886-020-01664-x

**Published:** 2020-10-02

**Authors:** Jennifer Fortuna, Anne Riddering, Linda Shuster, Cassie Lopez-Jeng

**Affiliations:** 1 Occupational Science and Therapy Department, Grand Valley State University, 500 Lafayette Ave NE, Grand Rapids, MI 49503 USA; 2 Department of Occupational Therapy, Western Michigan University, 1903 W. Michigan Ave, Kalamazoo, MI 49008 USA; 3 Department of Speech, Language and Hearing Sciences, Western Michigan University, 1903 W. Michigan Ave, Kalamazoo, MI 49008 USA; 4 School of Interdisciplinary Health Programs, Western Michigan University, 1903 W. Michigan Ave, Kalamazoo, MI 49008 USA

**Keywords:** Health literacy, Age-related macular degeneration, Patient education materials, Readability, Suitability

## Abstract

**Background:**

Age-related macular degeneration (AMD) is a chronic eye condition that leads to permanent vision loss in the central visual field. AMD makes reading challenging and inefficient. People with AMD often find it difficult to access, process and understand written patient education materials (PEMs). To promote health literacy, the demands of written PEMs must match the literacy capacities of the target audience. This study aims to evaluate the readability (grade level) and suitability (appropriateness) of online PEMs designed for people with AMD.

**Methods:**

Online PEMs were sourced from websites of national organizations providing patient education materials designed for people with AMD. The Flesch-Kincaid Grade Level formula and the Suitability Assessment of Materials instrument were used to assess the readability and suitability of PEMs. Descriptive statistics were used to compare online PEMs by organization based on national guidelines for readability level (≤ sixth grade) and the recommended suitability score (≥ 70%) for “superior” material.

**Results:**

One hundred online PEMs were evaluated from websites of 16 professional organizations. The mean readability level was 9.3 (range 5.0–16.6). The mean suitability score was 53% (range 18–78%). Only six (6%) of PEMs achieved the recommended guidelines for readability level and suitability score.

**Conclusion:**

The majority of online PEMs designed for people with AMD were written above the recommended readability level, and below the suggested suitability score. To promote health literacy, the demands of written health information must match the reading capacities of the target audience. Heeding to evidence-based guidelines for providing written information to patients with low health literacy and low vision is beneficial for both patients and health care providers. Future research is warranted.

## Background

Age-related macular degeneration (AMD) is a chronic eye condition that leads to permanent vision loss in the central visual field. AMD is a leading cause of vision loss for people age 50 and older [1]. An estimated 1.8 million people are affected by AMD in the United States (U.S.) alone [2]. Difficulty reading is one of the most common complaints from patients seeking low vision rehabilitation services [3, 4]. Central vision loss makes reading challenging and inefficient. Additional time, attention and effort are needed to process and understand written text [5]. Poorer reading performance may be due to decreased acuity and contrast sensitivity in the peripheral visual field, and factors associated with the size and style of font [6]. In low vision rehabilitation, techniques to increase reading performance are often addressed. Adaptations may include use of optical devices such as magnifiers and closed-circuit televisions (CCTVs), and eccentric viewing training which is learning to use the undamaged area of one’s vision. These interventions improve access to text; however, they do not increase processing or understanding of complex written information such as patient education materials (PEMs). To promote health literacy, the demands of written text must match the literacy capacities of the reader.

### Health literacy

Health literacy is defined as the degree to which individuals have the capacity to obtain, cognitively process and understand health information to make informed health-related decisions [7]. Health literacy is demonstrated through skills in basic literacy when reading and understanding health information. Low health literacy is a significant problem in the U.S. [8] According to the American Medical Association (AMA), over one-third of American adults, approximately 89 million people, have inadequate health literacy [9]. Health literacy is the single best predictor of health outcomes [9, 10].

### Readability and suitability

According to Legge, there are two reasons why reading comprehension may be poorer in people with low vision [11]. First, slower reading speed makes it difficult to maintain attention on text and integrate meaning across words and phrases. Second, the increased demands of decoding (i.e., translating print into words) and poorer quality of visual input may limit understanding. The readability and suitability of reading materials are additional factors that may impact reading performance in people with AMD. Readability is a quantitative assessment of the reading skills required to easily comprehend written material [10]. Readability is calculated by applying a mathematical formula to a sample passage of written text. A grade level (i.e., number of years of education needed to comprehend written text) is produced based on the number of syllables, words and sentences. Several formulas are used to assess readability; however, there is no consensus as to which formula is best to assess the readability of PEMs. The suitability (i.e., appropriateness) of written information is another important factor impacting comprehension of written health information [12]. For people with AMD, factors related to the layout and design of written information may support, or limit, comprehension of PEMs [11].

In 2016, the Program for the International Assessment of Adult Competencies (PIAAC) published the most current indicator of basic skills in literacy, numeracy and problem solving skills of American adults [13]. The PIAAC defines literacy as the ability to understand, evaluate, use and engage with written texts to participate in society, to achieve one’s goals and to develop one’s knowledge and potential. Findings from the survey indicated only 12% of American adults had proficient literacy skills. These results matched findings from the 2003 National Assessment of Adult Literacy (NAAL) survey which also found 12% of adult Americans had proficient health literacy skills to fully participate in the self-management of their own health [14]. According to the Centers for Disease Control and Prevention, people with low literacy are more likely to report poor health outcomes [15].

Comprehension of written health information is influenced by several factors including the ability to read text, locate and use written information in documents, and to use numbers embedded in print materials [16]. According to the Pfizer Principles for Clear Health Communication, health outcomes are impacted by low health literacy in two ways: (1) a mismatch between reading abilities and the reading level of written health information; and (2) lack of health-related information that is easy to understand [8]. Existing research indicates the impact of vision loss on health outcomes is often underestimated by health care providers [17, 18]. Health care providers who provide written PEMs must recognize how poor reading proficiency creates barriers to functional health literacy [19, 20].

The Center for Studying Health System Change reports 75% of physicians provide written PEMs on a routine basis [21]. Existing research has identified a discrepancy between PEM readability and the average American adult’s capacity to comprehend written health-related information [10, 22, 23]. Most PEMs are written at, or above, the tenth grade reading level and include written information too advanced for most patients to understand [24, 25]. On average, American adults read between the eighth and ninth grade level [14]. The gap is even wider for older adults. According to the United States Government Accountability Office, the average Medicare recipient reads at, or below, the fifth grade reading level [26]. The barriers to reading created by central vision loss put older adults with AMD at greater risk for low health literacy [14, 27]. To reach the needs of the largest range of adults, the AMA recommends health-related patient information be written below the sixth grade reading level [9]. For people with low literacy skills, the National Institutes of Health (NIH) Clear Communication Campaign suggests writing between the third and sixth grade reading level [28].

The internet has become the most widely accessible source of PEMs [29, 30]. A study by the Pew Internet and American Life Project found that 80% of American adults who use the internet have searched for online health information [31]. Although it has become easier to access PEMs online, most American adults are unable to process or understand the technical information within them to inform health-related decision making [29]. Determining whether existing PEMs meet the recommended guidelines for readability and suitability is a necessary first step for promoting health literacy and patient outcomes. This purpose of this study was to assess the general readability and suitability of online PEMs designed for people with AMD. This research is needed to determine if existing online PEMs are appropriate (i.e., readable and suitable) for this population, who is at greater risk for low health literacy [14, 27].

### Gaps in the literature

Existing research has explored the readability of PEMs across a variety of health conditions and subspecialties [10, 22, 23, 32–35]. A major gap in the literature exists surrounding treating people with AMD as a unique group under the larger umbrella of low vision [36]. A handful of studies have explored the readability of online PEMs for a range of different ophthalmic diagnoses [30, 37–39]. None of these studies have explored the readability of PEMs designed for people with AMD. The suitability (i.e., appropriateness) of PEMs is also important when determining the fit between written health-related information and the reading capacities of a target population. To date, this is the first study to focus solely on the readability and suitability of online PEMs designed for people with AMD. This study is needed to fuel future research and develop population-specific PEMs that meet the unique learning needs of this population.

### Purpose

The purpose of this study is to determine the general readability and suitability of online PEMs designed for people with AMD. Furthermore, this study aims to identify the percentage of online PEMs that achieve the national guidelines for readability level (≤ sixth grade) and the recommended suitability score (≥ 70%). The researchers hypothesize that the majority of online PEMs designed for people with AMD will be written at grade levels above the recommended readability level, and below the recommended suitability score. To date, this is the first study to assess the readability and suitability of online PEMs designed specifically for people with AMD.

## Methods

### Sample selection

This study was approved by the Institutional Review Board at Western Michigan University. A convenience sample of online PEMs was sourced from websites of professional organizations who provide patient education on AMD (Table 1). The primary researcher consulted with two occupational therapists specializing in low vision rehabilitation to identify credible sources of educational information. The target sample size for this study was 100 PEMs. To locate PEMs, the key words “age related macular degeneration (AMD)” were entered into the search engines of each organization’s website. If a search engine was not available, the primary researcher searched the website manually. To be included in the sample, PEMs had to meet the following inclusion criteria: (1) written by a professional society or clinical practice website; (2) published in English; (3) contain patient education designed for people with AMD. Scientific articles, opinion pieces, patient forums and PEMs about similar topics (e.g., low vision and Stargardt disease) were excluded from this study.

**Table 1:** Range and Mean of FKGL Readability Levels by Organization

	Institution/Organization	Total PEMs	FKGL Range	Mean FKGL Readability Level
1	NIH National Eye Institute	6	5.9–12.0	8.3
2	American Macular Degeneration Foundation	12	8.4–16.6	11.7
3	American Academy of Ophthalmology	5	5.3–11.0	7.4
4	NIH U.S. National Library of Medicine	4	5.0–6.8	6.1
5	UC Irvine Health MD Partnership	9	7.2–12.6	10.1
6	Foundation Fighting Blindness	4	10.4–13.3	11.7
7	American Printing House for the Blind	9	10.1–13.6	12.1
8	Prevent Blindness	5	7.5–10.6	8.5
9	Merck Manual Patient Education	2	5.9–10.3	8.1
10	Bright Focus Foundation	15	9.0–13.1	10.4
11	Mayo Foundation for Medical Education/Research	5	8.7–10.9	9.7
12	Macular Degeneration Foundation	11	6.8–14.6	9.7
13	Centers for Disease Control and Prevention	3	7.0–11.9	8.4
14	Macular Degeneration Support	2	8.0–9.9	9.0
15	Lighthouse Guild	2	8.3–8.4	8.4
16	Macular Society	6	7.6–9.9	8.6
Total/Mean Scores		*n* = 100	5.0–16.6	9.3

The Flesch-Kincaid Grade Level (FKGL) formula calculates readability (i.e., grade level) of written text based on average sentence length and syllables per word

### Procedures

One-hundred online PEMs were randomly selected from the websites of organizations providing patient education designed for people with AMD. The primary researcher determined the general readability and suitability of each individual PEM, as well as the percentage of PEMs that achieve the recommended readability (≤ sixth grade level) and suitability score (≥ 70%). Written text from each PEM was copied from the website and pasted into a Microsoft Word document [40]. To improve the accuracy of readability calculations, the text was cleaned prior to analysis. The process included removing all unrelated material such as copyright notices, disclaimers, date stamps, graphics, tables, author information, hyperlinks, in-text citations and reference lists. To achieve a uniform style, each passage of text was highlighted and “right-clicked” to access the “clear formatting” option. Next, bullets, paragraph breaks and some punctuation (e.g., quotation marks, parentheses, colons and semicolons) were removed. A period was added after each heading, sentence fragment or sentence. Numbers, decimals and percentages were converted to written form (e.g., “2.5%” was converted to “two point five percent”). To improve the accuracy of word count, dashes were removed and compound words were separated into root words (e.g., “age-related” was changed to “age related”).

### Flesch-Kincaid grade level (FKGL) formula

There are over 40 readability indices available to calculate the grade level of written text [41]. Each index utilizes a mathematical formula based on the number of syllables, complex words and sentences. Opinions vary as to which index is the most accurate; however, several readability formulas are used in health care settings [10]. The Flesch-Kincaid Grade Level (FKGL) formula measures readability of written text using the average sentence length and syllables per word [42]. The FKGL is a widely used readability formula [43, 44]. For this study, readability was calculated using the FKGL formula embedded in Microsoft Word software. To enable this tool, the researcher selected the “Review, Spelling & Grammar” functions in sequential order. A readability level is displayed after the grammar and spell check process is complete. The FKGL formula was chosen for this study because it is quick and easy to administer, has been extensively validated, and correlates highly with other readability formulas [10]. To assess the reliability of the FKGL tool embedded into Microsoft Word, 10 clean passages of text were randomly selected and entered into a second FKGL readability calculator available online [45]. There was near perfect agreement between the two readability calculators. The mean errors were 0.17 grade levels between the Microsoft Word and online FKGL readability calculators.

### Suitability assessment of materials (SAM)

When examining the match between the demands of written text and the capacities of the target audience, the impact of design characteristics on comprehension should also be considered. Factors such as graphics, layout and typography can be difficult to assess in an objective manner. The Suitability Assessment of Materials (SAM) instrument is a valid and reliable tool designed to assess the overall suitability (i.e., appropriateness) of health information for a specific audience [41]. The SAM has been administered successfully in previous research on health literacy [46–48]. For this study, the SAM instrument was used to measure the suitability of PEMs across six categories: (1) content; (2) literacy demand; (3) graphics; (4) layout and typography; (5) learning stimulation; and (6) cultural appropriateness. A percentage score and suitability rating was calculated for each PEM based on the appropriateness of health information for people with AMD. Interpretation of SAM scores are as follows: 0–39% - Not Suitable; 40–69% - Adequate; and, 70–100% - Superior [40]. Based on scoring and interpretation methods described by the authors, a SAM percentage score ≥ 70% is needed for PEMs to be considered suitable in this study.

### Data analyses

Statistical analysis was completed with IBM SPSS 25 software [49]. Descriptive statistics were used to determine mean FKGL and SAM scores and the percentage of online PEMs that achieved the national guidelines for readability level (≤ sixth grade) and recommended suitability score (≥ 70%).

## Results

One-hundred online PEMs were evaluated from 16 professional organizations providing patient education on AMD (Table 1). The range of reading levels varied across organizations. Based on results of the FKGL formula, the mean readability level was 9.3 (range 5.0–16.6). The majority (94%) of PEMs were written above the sixth grade reading level. Only six PEMs (6%) met the guidelines for readability level (≤ sixth grade) [9, 28]. Seventeen PEMs (17%) were written above the 12th grade reading level.

The suitability (i.e., appropriateness) of PEMs for the target population also varied across organizations. Results of the SAM instrument (Table 2) found a mean suitability score of 53% (range 18–78%), with a mean suitability rating of “adequate.” In total, 15 PEMs (15%) met the recommended suitability score (≥ 70%) for “superior” material. All six (100%) of the PEMs written below the sixth grade reading level fell into this category. Sixty two PEMs (62%) received a suitability rating of “adequate.” Twenty-three PEMs (23%) were rated “not suitable.” Thirteen of the 17 PEMs (76%) written at college reading level received a SAM rating of “not suitable.”

**Table 2:** SAM Suitability Ratings by Organization

	Institution/Organization	Total PEMs	Not Suitable	Adequate	Superior
1	NIH National Eye Institute	6	0 (0%)	4 (67%)	2 (33%)
2	American Macular Degeneration Foundation	12	8 (67%)	4 (33%)	0 (0%)
3	American Academy of Ophthalmology	5	0 (0%)	3 (60%)	2 (40%)
4	NIH U.S. National Library of Medicine	4	0 (0%)	1 (25%)	3 (75%)
5	UC Irvine Health MD Partnership	9	2 (22%)	6 (67%)	1 (11%)
6	Foundation Fighting Blindness	4	1 (25%)	3 (75%)	0 (0%)
7	American Printing House for the Blind	9	4 (44%)	5 (56%)	0 (0%)
8	Prevent Blindness	5	0 (0%)	3 (60%)	2 (40%)
9	Merck Manual Patient Education	2	0 (0%)	1 (50%)	1 (50%)
10	Bright Focus Foundation	15	0 (0%)	14 (93%)	1 (7%)
11	Mayo Foundation for Medical Education/Research	5	1 (20%)	3 (60%)	1 (20%)
12	Macular Degeneration Foundation	11	1 (9%)	10 (91%)	0 (0%)
13	Centers for Disease Control and Prevention	3	1 (33%)	2 (67%)	0 (0%)
14	Macular Degeneration Support	2	2 (100%)	0 (0%)	0 (0%)
15	Lighthouse Guild	2	0 (0%)	0 (0%)	2 (100%)
16	Macular Society	6	3 (50%)	3 (50%)	0 (0%)
Total/Mean Scores		*n* = 100	23 (23%)	62 (62%)	15 (15%)

Interpretation of SAM scores: 0–39% - Not Suitable; 40–69% - Adequate; 70–100% - Superior

## Discussion

The results of this study found the majority of online PEMs designed for people with AMD were written above the sixth grade reading level as suggested by the AMA and NIH [9, 28]. Existing research has identified a mismatch between the readability of existing PEMs and the reading and comprehension skills of American adults [9, 14]. Central vision loss creates a barrier to health literacy for people with AMD. Evidence-based guidelines for readability have been published by the AMA and the NIH [9, 28]. To reach the largest audience, PEMs should be written below the sixth grade reading level. Literacy demand (e.g., writing style, vocabulary and sentence construction) and the physical properties of text (e.g., font style and size, contrast, spacing) may create additional barriers to processing and understanding of written information [11]. Therefore, the suitability (i.e., appropriateness) of written health information should also be considered for specific target populations. The majority of PEMs included in this study did not achieve the recommended suitability score (≥ 70%) for “superior” material. Consideration must be given to the design characteristics of PEMs to determine if modifications are needed to promote health literacy in this population.

The American Printing House (APH) for the Blind Guidelines for Print Document Design offers helpful strategies for improving the readability and visibility of PEMs for people with low vision [50]. The APH guidelines include specific recommendations for document design including font style, white space, spacing and formatting of simple charts and graphics. At this time, there is no research on the effectiveness of APH guidelines for improving reading performance in people with low vision. Of the 100 PEMs included in this study, 98 were published several years after the APH guidelines were developed. Therefore, one could assume that most of the online PEMs included in this study have not been held to higher standards for people with low vision.

The suitability (i.e., appropriateness) of PEMs is equally as important for promoting health literacy for people with AMD. In this study, only six of the PEMs written at or below the sixth grade level received a suitability score (≥ 70%) for “superior” material. This finding shows that readability does not guarantee suitability. During data analysis, factors related to layout and typography (e.g., clutter, contrast, and graphics) significantly lowered the suitability score of PEMs with satisfactory readability level. For example, the readability levels of PEMs that received a SAM score of “not suitable” ranged between grade 7.8 and 16.6. In contrast, the readability levels of PEMs receiving “superior” SAM scores ranged between grade 5.0 and 10.7. Literacy demand was another important factor that should not be overlooked. Most of the PEMs included in this study provided general information on AMD; however, topics related to treatment and research often include unavoidable medical jargon. These PEMs had the highest readability levels and lowest suitability scores in the sample. Due to the need for patient education on these topics, this limitation may be unavoidable.

### Limitations

This study has limitations. This study did not provide the opportunity for people with AMD to evaluate the true readability and suitability of the PEMs in the sample. Therefore, the findings lack confirmation from people with AMD. This study is limited because a single readability index (e.g., the FKGL) was used to calculate the readability of PEMs. Furthermore, the FKGL is not a direct measure of comprehensibility; therefore, there is a possibility the results of this study could underestimate the level of difficulty required to read health information. Although the results of this study show the mean readability of the PEMs included in this study are higher than the recommended guidelines, additional factors should be considered. For example, the words “age-related macular degeneration” are considered difficult to read simply based on the number of syllables involved. These words appeared frequently and cannot be replaced. These limitations may impact the generalizability of results to the greater population of people with AMD.

### Future directions

The existing guidelines for promoting health literacy do not consider how age and visual impairment may create additional barriers to processing and understanding of written health information. Future research should address the need for treating people with AMD as a unique group under the larger umbrella of low vision. Chung questions whether reading performance could be enhanced by modifying certain characteristics of text to better match the capabilities of the peripheral visual system [6]. Additional studies are needed to determine the optimal design and presentation of PEMs for this population. Future research should also evaluate the benefits of PEMs that have been modified based on established guidelines for patients with low health literacy and low vision.

## Conclusion

The majority of online PEMs included in this study did not achieve the national guidelines for readability level (≤ sixth grade), or suitability score (≥ 70%). To promote health literacy in people with low health literacy and low vision, the demands of written health information must match the reading capacity of the target audience. Efforts should be made to improve the readability and suitability of PEMs designed for people with AMD. Providing PEMs patients can access, process and understand will promote health literacy and informed health-related decision making. Heeding to guidelines for patients with low health literacy and low vision is beneficial for patients and health care providers. Future research to explore the training needs of health care providers is warranted.

## Data Availability

All data generated or analyzed during this study are included in this published article. The datasets used and/or analyzed are available from the corresponding author on reasonable request.

## Abbreviations

AMD: Age-related macular degeneration
PEMs: Patient education materials
CCTV: Closed-circuit television
AMA: American Medical Association
NIH: National Institutes of Health
CDC: Centers for Disease Control
NAAL: National Assessment of Adult Literacy
FKGL: Flesch-Kincaid Grade Level formula
SAM: Suitability Assessment of Materials
APH: American Printing House for the Blind

## References

[1] National Eye Institute (2019). Age-related macular degeneration.

[2] Centers for Disease Control and Prevention (2015). Common eye disorders.

[3] Owsley C, McGwin G, Lee PP, Wasserman N, Searcey K (2009). Characteristics of low-vision rehabilitation services in the United States. Arch Ophthalmol.

[4] Rubin GS (2013). Measuring reading performance. Vis Res.

[5] Warren M, DeCarlo DK, Dreer LE (2016). Health literacy in older adults with and without low vision. Am J Occup Ther.

[6] Chung STL (2020). Reading in the presence of macular disease: a mini-review. Ophthalmic Physiol Opt.

[7] Ratzan SC, Parker RM (2000). Introduction. National Library of medicine current bibliographies in medicine: health literacy.

[8] Doak CC, Doak LG, Root JH. Assessing Suitability of Materials. Teaching Patients with Low Literacy Skills. 2nd ed. Philadelphia: JB Lippincott; 1996.

[9] Weiss BD (2007). Health literacy: a manual for clinicians.

[10] Badarudeen S, Sabbharwal S (2010). Assessing readability of patient education materials: current role in orthopaedics. Clin Orthop Rel Res.

[11] Legge GE. Psychophysics of Reading in Normal and Low Vision. NJ & London: Lawrence Erlbaum Associates; 2007.

[12] Wolf MS, King J, Wilson EA, Curtis LM, Bailey SC, Duhig J, Russell A, Bergeron A, Daly A, Parker RM, Davis TC, Shrank WH, Lambert B (2012). Usability of FDA-approved medication guides. J Gen Intern Med.

[13] Organization for Economic Cooperation and Development (OECD). OECD Skills Outlook 2013: First Results from the Survey of Adult Skills. France: OECD Publishing; 2013.

[14] Kutner M, Greenberg E, Jin C (2006). The health literacy of America’s adults: results from the 2003 National Assessment of adult literacy.

[15] Centers for Disease Control and Prevention (2019). Understanding literacy and numeracy.

[16] Rudd RE (2007). Health literacy skills of U.S. adults. Am J Health Behav.

[17] Chaudry I, Brown GC, Brown MM (2015). Medical student and patient perceptions of quality of life associated with vision loss. Can J Ophthalmol.

[18] Zhang S, Liang Y, Chen Y, Musch DC, Zhang C, Wang N (2015). Utility analysis of vision related quality of life in patients with glaucoma and different perceptions from ophthalmologists. J Glaucoma.

[19] Parker R (2000). Health literacy: a challenge for American patients and their health care providers. Health Promot Int.

[20] Warren M (2013). (2013). Promoting health literacy in older adults with low vision. Top Geriatr Rehabil.

[21] Carrier ERJ (2009). Expectations outpace reality: physicians’ use of care management tools for patients with chronic conditions. Issue Brief Center Stud Health Syst Change.

[22] Kher A, Johnson S, Griffith R. Readability assessment of online patient education material on congestive heart failure. Adv Prev Med. 2017;9780317. 10.1155/2017/9780317 PMC5471568 28656111

[23] Stossel LM, Segar N, Gliatto P, Fallar R, Karani R (2011). Readability of patient education materials available at the point of care. J Gen Intern Med.

[24] Davis TC, Crouch MA, Wills G, Abdehou DM (1990). The gap between patient reading comprehension and the readability of patient education materials. J Family Pract.

[25] Kirsch IS, Jungeblut A, Jenkins L, Kolstad A (1993). Adult literacy in America.

[26] United States Government Accountability Office [GAO] (2006). Medicare: Communications to beneficiaries on the prescription drug benefit could be improved.

[27] Harrison TC, Mackert M, Watkins CA (2010). Qualitative analysis of health literacy issues among women with visual impairments. Res Gerontol Nurs.

[28] National Institutes of Health (2018). Clear Communication: Clear & Simple.

[29] Armstrong-Heimsoth A, Johnson ML, Carpenter M, Thomas T, Sinnappan A (2019). Health management: occupational therapy’s key role in educating clients about reliable online health information. Open J Occup Ther.

[30] John AM, John ES, Hansberry DR, Prashant JT, Suqin G (2015). Analysis of online patient education materials in pediatric ophthalmology. J AAPOS.

[31] Fox S, Jones S (2011). The social life of health information. Pew Research Internet Project.

[32] D’Alessandro DM, Kingsley P, Johnson-West B (2001). The readability of pediatric patient education materials on the world wide web. Arch Pediat Adol Med.

[33] Eltorai AEM, Ghanian S, Adams CA, Born CT, Daniels AH (2014). Readability of patient education materials on the American Association for Surgery of trauma website. Arch Trauma Res.

[34] Hansberry DR, Agarwal N, Shah R, Schmitt PJ, Baredes S, Setzen M, Carmel PW, Prestigiacomo CJ, Liu JK, Eloy JA (2013). Analysis of readability of patient education materials from surgical subspecialties. Gene Otolaryngol.

[35] John AM, John ES, Hansberry DR, Lambert WC (2016). Assessment of online patient education materials from major dermatologic associations. J Clin Aesthet Dermatol.

[36] Beverly CA, Bath PA, Booth A (2004). Health information needs of visually impaired people: a systematic review of the literature. Health Soc Care Community.

[37] Edmunds MR, Barry RJ, Denniston AK (2013). Readability assessment of online ophthalmic patient information. JAMA Ophthalmol..

[38] Huang G, Fang CH, Agarwal N, Bhagat N, Eloy JA, Langer PD (2015). Assessment of online patient education materials form major ophthalmologic associations. JAMA Ophthalmol.

[39] John ES, John AM, Hansberry DR, Patel C (2014). Readability assessment of online ophthalmology information – a comprehensive comparison of education resources. Invest Ophthalmol Vis Sci.

[40] Word Software. Microsoft. 2016. https://www.microsoft.com/en-us/microsoft-365/word. Accessed 24 Mar 2020.

[41] Doak CC, Doak LG, Root JH. Assessing suitability of materials. Teaching patients with low literacy skills. 2nd ed: JB Lippincott; 1996.

[42] Kincaid JP (1975). Derivation of new readability formulas for navy enlisted personnel.

[43] Albright J, de Guzman C, Acebo P, Paiva D, Faulkner M, Swanson J (1996). Readability of patient education materials: implications for clinical practice. Appl Nurs Res.

[44] Cooley ME, Moriarty H, Berger MS, Selm-Orr D, Coyle B, Short T (1995). Patient literacy and the readability of written cancer educational materials. Oncol Nurs Forum.

[45] Edit Central (2020). Readability calculator.

[46] Eames S, McKenna K, Worrall L, Read S (2003). The suitability of written education materials for stroke survivors and their carers. Top Stroke Rehabil.

[47] Taylor-Clarke K, Henry-Okafor Q, Murphy C, Keyes M, Rothman R, Churchwell A, Mensah GA, Sawyer D, Sampson UKA (2012). Assessment of commonly available educational materials in heart failure clinics. J Cardiovasc Nurs.

[48] Weintraub D, Maliski SL, Fink A, Choe S, Litwin MS (2004). Suitability of prostate cancer education materials: applying a standardized assessment tool to currently available materials. Patient Educat Couns.

[49] SPSS Software. IBM. 2019. https://www.ibm.com/analytics/spss-statistics-software. Accessed 24 Mar 2020.

[50] Kitchel JE (2011). APH guidelines for print document design. American Printing House for the Blind.

